# The Effects of the Passage of Time from the 2011 Tohoku Earthquake on the Public’s Anxiety about a Variety of Hazards

**DOI:** 10.3390/ijerph13090866

**Published:** 2016-08-31

**Authors:** Kazuya Nakayachi, Kazuhisa Nagaya

**Affiliations:** 1 Faculty of Psychology, Doshisha University, Kyotanabe-shi, Kyoto 610-0394, Japan; 2 Graduate School of Psychology, Doshisha University Kyotanabe-shi, Kyoto 610-0394, Japan; karuken34@gmail.com

**Keywords:** the Tohoku Earthquake, the Fukushima Daiichi Nuclear Power Plant, anxiety about hazards, time passage

## Abstract

This research investigated whether the Japanese people’s anxiety about a variety of hazards, including earthquakes and nuclear accidents, has changed over time since the Tohoku Earthquake in 2011. Data from three nationwide surveys conducted in 2008, 2012, and 2015 were compared to see the change in societal levels of anxiety toward 51 types of hazards. The same two-phase stratified random sampling method was used to create the list of participants in each survey. The results showed that anxiety about earthquakes and nuclear accidents had increased for a time after the Tohoku Earthquake, and then decreased after a four-year time frame with no severe earthquakes and nuclear accidents. It was also revealed that the anxiety level for some hazards other than earthquakes and nuclear accidents had decreased at ten months after the Earthquake, and then remained unchanged after the four years. Therefore, ironically, a major disaster might decrease the public anxiety in general at least for several years.

## 1. Introduction

People sometimes worry about trivial risks while they sometimes underrate serious risks. Since its early stages, risk perception research has been seeking to identify factors that influence the public’s responses to risks [1,2,3]. Among a variety of factors, an occurrence of a catastrophic disaster and the passage of time after the event will influence the public’s anxiety regarding a variety of hazards. However, catastrophes cannot be manipulated by researchers and a very long time is needed to verify the effects of the passage of time after these events. For these reasons, the effects of the passage of time after a catastrophic disaster have not been sufficiently reported. This research examines these effects, investigating whether people’s anxiety about a variety of hazards has changed according to the passage of time after the 2011 Tohoku Earthquake.

The affect heuristic is a mental shortcut for the judgment of risk. People tend to base their judgment on what they feel about the risk rather than on what they think about it [4,5]. The “risk as feelings” hypothesis, also emphasizes the role of affect experienced in decision-making processes, rather than cognitive calculation of the desirability and likelihood of possible outcomes of choices [6,7]. Following the proposals of these models, several empirical reports have demonstrated that affect plays a central role in determining the perception of and behavior toward risks [8,9,10,11,12,13,14]. This study focused on anxiety in line with this stream of research.

### 1.1. The Enormous Damage Caused by the Tohoku Earthquake and Changes in Anxiety

A colossal earthquake of momentous magnitude (*Mw*) 9.0 struck the northeastern region of Japan on 11 March 2011 [15]. The human loss totaled over 18,000 with approximately 16,000 confirmed dead and the rest missing [16]. Many of these casualties resulted from the tsunami, rather than buildings collapsing or fires due to the earthquake. There were more than 400,000 homes damaged or destroyed and approximately 330,000 people were displaced and forced to take long-term refuge. The economic loss for Japan as a whole has been massive, at an estimated 16 trillion yen (approximately US$150 billion) [17].

Furthermore, the Tohoku Earthquake caused a serious accident at the Fukushima Daiichi Nuclear Power Plant. The incident was designated as Level 7, which is the highest level on the International Nuclear Event Scale (INES) [18]. After the accident, the cumulative exposure to radiation in a vast area around Fukushima Prefecture is considered to be more than 5 millisieverts (mSv) per year [19]. The annual average dose of exposure in Japan is approximately 2.1 mSv, therefore the accident more than doubled this amount [20]. Habitation has become restricted in the 20 km radius around the Fukushima Daiichi Nuclear Power Plant as well as anywhere where the expected cumulative radiation exposure is more than 20 mSv per year. Radioactive substances scattered and spread over a vast area in the Kanto and Tohoku regions of Japan contaminating its soil, rivers, and the ocean, and restrictions were put in place with respect to the consumption of agricultural and marine food products that exceeded the maximum allowable level of radiation.

After experiencing such serious disasters, it would be unsurprising if the Japanese public felt considerable anxiety toward earthquakes and nuclear power plants. Before and after the disaster, Nakayachi, Yokoyama, and Oki [21] conducted nationwide surveys with highly representative random samples to measure the public’s level of anxiety toward various hazards. The analysis revealed that anxiety toward earthquakes and nuclear power plants indeed increased after the disaster. Simultaneously, the analysis made clear that the trust in risk-managers relating to earthquakes and nuclear power plants had also decreased markedly [22]. Of particular interest was the finding that people’s anxiety toward hazards other than earthquakes had also changed. There were three possible reasons why anxiety toward other hazards could have changed. The first possibility was that there would be an increase in anxiety toward other natural disasters and the use of science and technology. Johnson and Tversky [23] showed that, after reading messages about certain risks, risk perception increases not only for that target risk but for other risks as well. The affective network model posits that mental representations of various events are tied together by emotions [24,25]. According to this model, increases in negative feelings attached to a hazard, such as earthquakes, are usually shared with other associated hazards, such as those towards natural disasters. The second possibility was that the public evaluates each hazard independently, and therefore earthquakes and nuclear power plant accidents do not influence the evaluation of other hazards. The third possibility was that anxiety toward other hazards would decrease because people have a finite ability to worry: by focusing their worries on earthquakes and nuclear plant disasters, due to limited attentional resources, anxiety regarding other hazards would be diminished. The model that proposes there are limitations on how much one can worry is based on the finite-pool-of-worry (FPW) hypothesis [26,27]. The findings of Nakayachi et al. [21] confirmed the third possibility. After the disaster, the only increases in anxiety were observed toward earthquakes, nuclear disasters, and crises of the national pension plan; overall, the level of anxiety regarding the other hazards had decreased.

### 1.2. The Passage of Time after the Tohoku Earthquake

A few years have passed without any major earthquakes or any nuclear power plant accidents. How much has the public’s anxiety regarding earthquakes and nuclear power plants diminished with the passage of time? Furthermore, in what way, if any, has there been a change in the degree of anxiety regarding other hazards? To approach these questions, we conducted a survey four years after the disaster and compared its results to those of past surveys.

To investigate how the passage of time changes the public’s anxiety after any major disaster, it is essential that no new disasters of the same kind have taken place in the period of interest. While neither massively destructive earthquakes nor nuclear power plant disasters struck Japan in the five-year period after the Tohoku Earthquake, in April 2016, Japan was hit by another major destructive earthquake, referred to as the 2016 Kumamoto Earthquake. On 14 April a 6.2 *Mw* earthquake struck, followed on 16 April by another major earthquake (7.0 *Mw*) [28]. The death toll was 55 individuals, 1814 people were injured, over 160,245 homes were damaged, and at its peak, there were over 180,000 people taking refuge [29]. Therefore, the survey conducted four years after the Tohoku Earthquake and before the 2016 Kumamoto Earthquake—which we report in this paper—to examine the influence of the passage of time in a period of relative calmness (in terms of earthquakes and nuclear power plant related catastrophes) actually coincided with a rare window of opportunity to conduct such a study.

### 1.3. Hypothesis

Between the Tohoku Earthquake of March 2011 and the Kumamoto Earthquake of April 2016, there was no major damage caused by any earthquakes. In addition to the years of calmness, the gambler’s fallacy might affect public anxiety about the earthquake [30]. If people experience a less frequent event, they tend to think that they will not experience the event for a long time thereafter. The decommissioning of the Fukushima Daiichi Nuclear Power Plant, while encountering difficulties, progresses nonetheless, and no other nuclear power plant related accidents have exposed the residents to radiation. Accordingly, we hypothesized that the level of anxiety toward earthquakes and nuclear power plants, which increased following the Tohoku Earthquake of 2011, would have decreased in the intervening four years. As negativity bias [31] and the asymmetry principle of trust [32] indicate, if one’s evaluation of a certain subject decreases, it is not easy to recover or regain what has been lost. Therefore, it is also possible that even after several years of calm, anxiety levels may not have returned to the same level as before the disaster. In contrast, with respect to earthquakes and nuclear power plants, we could find no reason that would increase the anxiety level beyond what was expressed after the Tohoku Earthquake. Thus, we hypothesized that anxiety toward these two hazards would have decreased compared to immediately after the earthquake.

Furthermore, we also examined how anxiety toward other hazards changed. If we simply apply the FPW hypothesis [26,27]—which hypothesizes that there is a finite amount of anxiety a person can appreciate—then anxiety regarding earthquakes and nuclear power plants would trade-off anxieties regarding other hazards. Therefore, if anxiety toward earthquakes and nuclear power plants decreased toward the pre-disaster level, then anxiety over other matters, which had dropped following the disaster, would necessarily increase. However, as we detail below, there is evidence against this proposal.

In one unimaginably destructive event, the Tohoku Earthquake impressed the public with a strong psychological impact [33], which increased anxiety toward earthquakes and nuclear power plants. Concurrently, anxiety regarding other hazards decreased in a counterbalancing response [21]. In short, a drastic event could decrease anxiety regarding other hazards. Yet what would decrease anxiety toward earthquakes and nuclear power plants is something less dramatic—the uneventful and hardly noticeable passage of time. No drastic, positive event exists that could rival the impact of the great earthquake and the immense damage which occurred exceptionally rapidly. Therefore, one might hypothesize that even if anxiety toward earthquakes and nuclear power plants decreased over time, this would occur slowly, and there would not be a notable counterbalancing increase in anxiety toward other hazards.

No study to date has empirically examined long-term changes in anxiety caused by major disasters, including the hazard central to the disaster along with its influence on anxiety regarding other hazards. We approached this subject through a survey which included a highly representative participant sample covering all of Japan.

## 2. Materials and Methods

### 2.1. Questionnaires

The hazard items in the questionnaire were the same as those in the 2008 and 2012 surveys [21]. Participants answered questions assessing their level of anxiety toward each of 51 types of hazard using a six-point Likert scale, where 0 = *have absolutely no anxiety* and 5 = *have great anxiety*. The survey items were selected based on commonly listed hazards in prior studies of risk perception. The items sampled diverse categories, including natural disasters, such as earthquakes and typhoons; accidents caused by the use of technology, such as nuclear power plant accidents and railway accidents; items categorized as chemical science, such as nanotechnology and pesticides; items relating to broad environmental hazards, such as global warming and environmental pollution by chemicals; crime related items, such as personal offenses and property offenses; international conflict-related items, such as war and terrorism; and disease-related items such as AIDS and BSE (bovine spongiform encephalopathy, commonly referred to as mad cow disease). In addition, we included items that have become major social issues in Japan over the past two decades, such as falsification of quake-capacity of residential buildings, mislabeling of food items, the crisis of the national pension plan, and child abuse. In selecting the items, we covered a wide variety of items while limiting the number of items, so as to not overburden the respondents and thus improve data quality. The 51 survey items are listed in the Results section.

The questions addressed in this paper constitute a portion of a larger research project. However, the questions evaluating the level of anxiety toward the 51 hazards were presented before all other questions and therefore there were no carry-over effects from the other questions that were asked.

### 2.2. Participants

The participants were pooled using two-phase stratified sampling based on region and city size. This method is the same as in previous surveys [21]. As the first phase, the survey locations were randomly selected from all over Japan by first calculating the number of survey points according to the population of each regional category. In Japan, every local government maintains a Basic Resident Register which contains every resident’s information such as name, address, gender, and date of birth. Use of these for academic research purposes is usually approved. As the second phase, using the Basic Resident Register of each selected location, we randomly chose a predetermined number of adults over 20 years of age. This sampling procedure was conducted for each survey and the participants were all different.

In the pre-quake 2008 survey, 53.6% of the sampled individuals (*N* = 1192) responded to the survey, of whom 52.0% (*n* = 620) were female and 48% (*n* = 572) were male. The age distributions were as follows: 12.2% (*n* = 145) were in their 20s, 16.0% (*n* = 191) were in their 30s, 18.5% (*n* = 221) were in their 40s, 21.6% (*n* = 257) were in their 50s, 18.9% (*n* = 225) were in their 60s, and 12.8% (*n* = 153) were in their 70s or older.

In the survey conducted 10 months after the quake in 2012, 56.9% of individuals (*N* = 1138) responded, of whom 52.9% (*n* = 602) were female and 47.1% (*n* = 536) were male. The age distributions were as follows: 9.4% (*n* = 107) were in their 20s, 14.9% (*n* = 169) were in their 30s, 15.0% (*n* = 170) were in their 40s, 19.9% (*n* = 227) were in their 50s, 22.8% (*n* = 260) were in their 60s, and 18.0% (*n* = 205) were in their 70s or older.

In the survey conducted in 2015, four years after the quake, 53.6% of individuals (*N* = 1073) responded, of whom 49.5% (*n* = 602) were female and 50.5% (*n* = 536) were male. The age distributions were as follows: 9.9% (*n* = 106) were in their 20s, 15.0% (*n* = 161) were in their 30s, 20.8% (*n* = 223) were in their 40s, 17.0% (*n* = 182) were in their 50s, 20.3% (*n* = 218) were in their 60s, and 17.1% (*n* = 183) were in their 70s or older.

### 2.3. Procedure

The pre-quake survey was conducted in January 2008. The first post-quake survey was conducted in January 2012, 10 months after the disaster. The second post-quake survey was conducted in February 2015, four years after the disaster. Surveyors visited the home of the respondents, provided the questionnaires, and at a later date revisited those homes to collect the completed questionnaires. They checked through the answers when they collected the completed questionnaires. The participants were given a 500-yen (approximately US$5) gift card redeemable for books as an incentive. All subjects gave their informed consent for inclusion before they participated in the study. The protocol of this study was approved by the Ethics Committee of Doshisha University (Project identification code: 14072).

## 3. Results

Table 1 shows the average anxiety ratings by hazard at each survey. The mean of missing data by item was 0.41% (standard deviation: *SD* = 0.21) with the range from 0.12% (property offenses) to 1.56% (nanotechnology). Missing data were deleted from the calculations. The hazard items were sorted in descending order of scores in the 2015 survey. Earthquake ranked highest on the anxiety scale in all three surveys. The score increased from the 2008 survey to the 2012 survey, then declined in 2015 to almost the same level as pre-quake. With the exception of earthquake, nuclear plant accident (fifth in 2015) in the 2012 survey was the only item that recorded an average score of 4.0 and above on the five-point scale ranging from 1 to 5. The shift from the 2008 survey to the 2012 and 2015 surveys was similar to that of earthquake. It increased drastically for a time after the Tohoku Earthquake, and then decreased over four years. The average scores of “falsification of quake-capacity” (24th place), an item related to earthquake, were not high and they showed no increase in anxiety in the 2012 and 2015 surveys. Severe earthquakes sometimes cause conflagrations. However, there was no increase in anxiety scores about “residential fire” (20th place) and “office building fire” (41st place). Severe earthquakes also cause injury by “falling” (43rd place). Again, the scores remained relatively low across the surveys.

A two-way (period × hazard item) analysis of variance (ANOVA) using anxiety score as the dependent variable was performed. Listwise deletion was applied to the missing data. The results revealed significant main effects of period (*F*(2, 3082) = 10.68, *p* < 0.001, partial η^2^ = 0.007) and hazard items (*F*(50, 154,100) = 704.05, *p* < 0.001, partial η^2^ = 0.186), and a significant interaction (*F*(100, 18,540) = 17.46, *p* < 0.001, partial η^2^ = 0.11). As the interaction was significant, the simple main effects of period were tested for each hazard item. Table 2 shows the results along with the changes in anxiety scores of each hazard between the two consecutive surveys. The hazard items were sorted in descending order of changes in scores from the 2008 survey to the 2012 survey. Values in bold indicate significant differences in score between the two surveys. The item that increased most in score from pre-quake to 10 months post-quake was “nuclear plant accident” (first place) followed by “earthquake” (second place). Then the scores of both nuclear plant accident and earthquake significantly reduced from 10 months post-quake to four years post-quake.

Of the hazards other than earthquake and nuclear plant accident, “crisis of national pension plan” was the only item that showed a significant rise in anxiety from pre-quake to 10 months post-quake. Of the remaining 48 items, 27 showed significant drops in anxiety ratings and 21 did not change significantly. Among the 27 hazards that changed, there were many related to food issues (agrochemicals, chemical food additives, genetically modified organism (GMO), mislabeling of food, natural food additives, BSE, and alcohol), to so-called environmental problems in the broad sense (global warming, indoor chemicals, asbestos, oil depletion, tobacco, endocrine disruptor, dioxin, environmental pollution by chemicals, and abnormal weather) and to medical issues (new infectious diseases, medical malpractice, medicinal side effects, and AIDS). For these 27 items, there were no hazards that significantly increased in anxiety score from 10 months post-quake to four years post-quake, with the exception of terrorism and airplane accidents. That is, while anxiety regarding these 27 hazards had decreased in a counterbalancing response to the increase of anxiety over nuclear plant accidents and earthquakes at 10 months after the Tohoku Earthquake, no counterbalancing response was observed for the decrease of anxiety over nuclear plant accidents and earthquakes four years after the earthquake, with two exceptions. These results provide support for the hypothesis.

Among the 21 hazards that did not show a change, there were many causes of death that cost thousands of lives annually (cancer, brain and heart diseases, lifestyle-related illnesses, traffic accidents, suicide, and suffocation by food) and hazards related to earthquakes (falsification of quake-capacity, falling, residential fires, and office building fires). Again, no increases in anxiety related to any hazard were observed in the four years post-quake survey. The significant decrease in anxiety about unemployment was consistent with the decrease of the unemployment rate in Japan [34].

As a whole, the passage of four years after the Tohoku Earthquake caused little change in anxiety regarding a variety of hazards other than earthquakes and nuclear plant accidents. There were no increases in anxiety related to any hazards from the 2012 survey to the 2015 survey, with the exceptions of terrorism and airplane accidents. Especially, the anxiety about terrorism has increased markedly. The explanation for this will be provided in the Discussion.

## 4. Discussion

The massive earthquake that occurred in the Tohoku region of Japan in 2011 claimed many lives, caused many casualties, and engendered major economic loss. Through a chain of events triggered by this earthquake, the Fukushima Daiichi Nuclear Power Plant underwent a serious accident categorized as level 7 of the INES system, and radioactive substances contaminated a vast area. Through this disaster, the anxiety Japanese people felt toward a variety of hazards changed. The changes that were reflected in the results of surveys can be summarized as follows:

The anxiety toward earthquakes and nuclear power plant accidents increased immediately after the Tohoku Earthquake. However, in the four years that followed, the level of anxiety toward earthquakes decreased to its original, pre-disaster level, and anxiety toward nuclear power plant accidents also underwent a major decline. In contrast, public anxiety regarding a variety of hazards and events, especially those related to food issues, so-called environmental problems, and medical issues, decreased immediately after the earthquake, and remained at a low level for several years. These hazards are not relevant to the Tohoku Earthquake, which was a topical unprecedented disaster associated with a severe technological accident. These results indicate that such catastrophes have the ironic effect of reducing the anxiety the public has regarding other hazards.

With respect to anxieties not only over causes of death that cost many lives annually (e.g., cancer) but also over some hazards relevant to earthquakes (e.g., falsification of quake-capacity and residential fires), the 2011 Tohoku Earthquake had no influence at all. Why did the anxiety about such hazards relevant to earthquakes not increase? The reason might reflect how the Tohoku Earthquake took residents’ lives. As mentioned in the Introduction, many of the casualties resulted from the tsunami, rather than buildings collapsing or fires due to the earthquake. For that reason, hazards that may produce deaths by collapsing and fires did not increase even though they could cause many casualties in other types of earthquake.

There were no increases in anxiety scores related to any hazards in 2015, except for scores related to terrorism and airplane accidents. Especially, anxiety over terrorism markedly increased. The extent of change in score was the second largest following that of nuclear plant accidents observed 10 months after the Tohoku Earthquake. The reason for the upsurge in anxiety about terrorism seems to be obvious. Immediately before the survey period, two Japanese, who had been kidnapped by Islamic State (IS) for several months, were killed with a sword. This was the first case of Japanese people being killed by IS. The videos of the two hostages with a member of IS with a sword in his hand were on air repeatedly along with many news reports of terrorists’ suicide bombings. It is considered that these cruel and horrible events frightened Japanese people at the time of our third survey.

As mentioned in the Introduction, affect is one of the main issues in the current research on risk perception. However, few longitudinal studies have reported changes in public anxiety regarding a variety of hazards after a catastrophic disaster. Therefore, the survey results of this article will provide basic data for future research. The findings from the data in this study will also contribute to the theoretical development of comprehensive understanding of public risk perception.

This research, however, has some limitations that need to be mentioned before discussing potential future research. Even though the surveys in this research used highly representative samples, they are only representative of the Japanese population. It would be desirable to implement this survey in other countries, to examine the generalizability of our findings. This research focused on shifts in public anxiety at the macro level. Individual difference variables, such as socioeconomic status, direct exposure to hazards, and personal experiences like post-traumatic stress disorder, were not measured. There is also another limitation in this research. The last survey reported in this article was conducted four years after the 2011 Tohoku Earthquake. What happens to anxiety regarding hazards over an extended passage of time is an interesting research question. However, in April 2016, the Kumamoto Earthquake occurred. For this reason, it is no longer possible to test the impact of an uneventful passage of time where no earthquake-related disasters have occurred after the Tohoku Earthquake. Note that although the Kumamoto Earthquake caused considerable damage, its scale was much smaller than that of the Tohoku Earthquake, and there were no nuclear power plant accidents or radiation-related contamination. From this perspective, how this additional disaster influenced anxiety toward hazards is also of interest. While the Kumamoto Earthquake was a major earthquake, it caused less damage than the Tohoku Earthquake, so it is possible that anxiety regarding earthquakes may not have increased subsequently. Conversely, the public experienced two colossal earthquakes in the span of just five years, and this short interval may have heightened the anxiety level of the public more than the extent of damage the quakes caused. Recently, McClure et al. [30] found that the two earthquake sequences were having a lasting impact, particularly in the area near where the second earthquake occurred. Furthermore, as this major earthquake in Kumamoto took place in the southwest region of Japan, far from the northeastern region where the Tohoku Earthquake occurred, the Japanese population may have been reminded that in Japan, a major earthquake can strike anywhere. Consequently, the anxiety over operating a nuclear power plant in this country may have increased. Alternatively, given that there were no nuclear power plant accidents associated with this recent earthquake, the anxiety level might continue to decrease. To answer these questions it would be necessary to continue to collect data on people’s assessments of various hazards.

## 5. Conclusions

This study revealed the effects of the 2011 Tohoku Earthquake and several years’ passage of time with no severe earthquakes and nuclear accidents on the public anxiety about a variety of hazards. The anxiety toward earthquakes and nuclear power plant accidents increased immediately after the Tohoku Earthquake. However, in the four years that followed, the level of anxiety toward earthquakes decreased to its original, pre-disaster level, and anxiety toward nuclear power plant accidents also underwent a major decline. In contrast, public anxiety regarding a variety of hazards and events, especially those related to food issues, so-called environmental problems, and medical issues, decreased immediately after the earthquake, and remained at a low level for several years. These results indicate that catastrophic disasters have the ironic effect of reducing the anxiety the public has regarding other hazards.

## Figures and Tables

**Table 1:** Average anxiety ratings for each hazard measured in the surveys in 2008, 2012, and 2015.

Ranking Order	Item	2015 Survey	2013 Survey	2008 Survey
		Mean (SD)	Mean (SD)	Mean (SD)
1	Earthquake	4.00 (1.14)	4.24 (0.99)	3.99 (1.14)
2	Cancer	3.79 (1.25)	3.82 (1.22)	3.81(1.22)
3	Crisis of national pension plan	3.78 (1.30)	3.95 (1.18)	3.74 (1.35)
4	Traffic accident	3.67 (1.14)	3.71 (1.17)	3.75 (1.12)
5	Nuclear plant accident	3.64 (1.44)	4.10 (1.25)	3.34 (1.37)
6	Brain and heart disease	3.61 (1.25)	3.63 (1.22)	3.61 (1.23)
7	Terrorism	3.60 (1.40)	2.91 (1.53)	3.20 (1.46)
8	War	3.55 (1.41)	3.43 (1.47)	3.47 (1.44)
9	Global warming	3.51 (1.26)	3.58 (1.20)	3.98 (1.06)
10	New infectious disease	3.47 (1.33)	3.59 (1.25)	3.76 (1.28)
11	Personal offenses	3.45 (1.37)	3.44 (1.37)	3.60 (1.32)
12	Environmental pollution by chemicals	3.44 (1.27)	3.56 (1.23)	3.71 (1.16)
13	Abnormal weather	3.40 (1.29)	3.46 (1.28)	3.63 (1.22)
14	Property offenses	3.34 (1.21)	3.35 (1.27)	3.47 (1.23)
15	Lifestyle-related illness	3.26 (1.21)	3.27 (1.22)	3.34 (1.23)
16	Typhoon	3.25 (1.29)	3.21 1.247	3.15 (1.35)
17	Chemical food additives	3.24 (1.29)	3.22 (1.29)	3.63 (1.20)
18	Child abuse	3.24 (1.47)	3.27 (1.50)	3.33 (1.44)
19	Medical malpractice	3.22 (1.37)	3.33 (1.34)	3.55 (1.22)
20	Residential fire	3.19 (1.32)	3.29 (1.32)	3.32 (1.32)
21	Mislabeling of food	3.17 (1.38)	3.25 (1.37)	3.59 (1.31)
22	Bullying in school	3.13 (1.51)	3.03 (1.51)	3.20 (1.49)
23	Medicinal side effect	3.11 (1.35)	3.17 (1.32)	3.41 (1.33)
24	Falsification of quake-capacity	3.06 (1.43)	3.20 (1.43)	3.22 (1.42)
25	Missile strike	2.98 (1.51)	2.98 (1.50)	2.99 (1.47)
26	Dioxin	2.92 (1.39)	2.99 (1.38)	3.20 (1.38)
27	Oil depletion	2.92 (1.37)	3.29 (1.33)	3.57 (1.27)
28	Fire from home appliance	2.90 (1.36)	2.86 (1.40)	2.97 (1.40)
29	Unemployment	2.89 (1.67)	3.24 (1.66)	3.13 (1.65)
30	Agrochemicals	2.86 (1.40)	2.94 (1.39)	3.43 (1.34)
31	Water accident	2.77 (1.43)	2.76 (1.44)	2.83 (1.41)
32	GMO	2.74 (1.38)	2.73 (1.40)	3.09 (1.32)
33	Tobacco	2.73 (1.59)	2.77 (1.56)	3.04 (1.50)
34	Ultraviolet rays	2.73 (1.34)	2.80 (1.34)	3.00 (1.30)
35	Asbestos	2.64 (1.56)	2.77 (1.54)	3.08 (1.50)
36	Endocrine disruptor	2.61 (1.35)	2.72 (1.34)	2.95(1.33)
37	Lightning	2.61 (1.45)	2.58 (1.44)	2.59 (1.49)
38	Railway accident	2.60 (1.42)	2.75 (1.41)	2.71 (1.37)
39	Airplane accident	2.57 (1.51)	2.40 (1.50)	2.63 (1.50)
40	AIDS	2.52 (1.59)	2.63 (1.61)	2.90 (1.59)
41	Office building fire	2.52 (1.41)	2.60 (1.47)	2.71 (1.45)
42	BSE	2.52 (1.46)	2.75 (1.51)	2.99 (1.40)
43	Falling	2.49 (1.47)	2.47 (1.42)	2.45 (1.43)
44	Suffocation by food	2.34 (1.50)	2.31 (1.50)	2.24 (1.51)
45	Indoor chemicals	2.31 (1.33)	2.34 (1.30)	2.70 (1.30)
46	Nanotechnology	2.27 (1.41)	2.26 (1.44)	2.45 (1.39)
47	Suicide	2.24 (1.62)	2.32 (1.61)	2.32 (1.64)
48	Natural food additives	2.15 (1.32)	2.17 (1.35)	2.47 (1.39)
49	Alcohol	2.06 (1.44)	2.09 (1.52)	2.24 (1.45)
50	Accidental poisoning by home boiler	2.06 (1.47)	2.12 (1.49)	2.40 (1.49)
51	Domestic discord	1.98 (1.56)	2.03 (1.55)	2.07 (1.55)

Note: Items were sorted in descending order of scores in the 2015 survey. BSE refers to bovine spongiform encephalopathy (commonly referred to as mad cow disease) and GMO refers to genetically modified organism.

**Table 2:** The results of the tests of simple main effect and mean differences in anxiety score between the two surveys.

Ranking Order	Item	Simple Main Effect			Difference	95% CI		Difference	95% CI	
		*F*	*p*	Partial η^2^	2015–2012	LL	UL	2012–2008	LL	UL
1	Nuclear plant accident	84.49	<0.001	0.052	**−0.473**	−0.617	−0.329	**0.760**	0.618	0.901
2	Earthquake	16.59	<0.001	0.011	**−0.225**	−0.342	−0.109	**0.252**	0.137	0.366
3	Crisis of national pension plan	7.83	<0.001	0.005	**−0.195**	−0.332	−0.059	**0.190**	0.056	0.323
4	Unemployment	10.78	<0.001	0.007	**−0.338**	−0.514	−0.162	0.130	−0.042	0.302
5	Suffocation by food	1.35	0.26	0.001	0.046	−0.113	0.206	0.062	−0.095	0.218
6	Typhoon	1.97	0.14	0.001	0.070	−0.068	0.208	0.043	−0.092	0.179
7	Railway accident	2.92	0.06	0.002	−0.143	−0.292	0.005	0.033	−0.112	0.179
8	Suicide	0.73	0.48	0.000	−0.084	−0.256	0.087	0.024	−0.145	0.192
9	Brain and heart disease	0.08	0.93	0.000	−0.017	−0.148	0.114	0.019	−0.109	0.148
10	Falling	0.59	0.56	0.000	0.060	−0.093	0.213	0.001	−0.150	0.150
11	Falsification of quake-capacity	3.41	0.03	0.002	−0.143	−0.295	0.009	−0.001	−0.150	0.148
12	Cancer	0.12	0.89	0.000	−0.020	−0.150	0.111	−0.006	−0.134	0.122
13	Missile strike	0.04	0.96	0.000	0.019	−0.140	0.179	−0.012	−0.168	0.144
14	Lightning	0.26	0.77	0.000	0.046	−0.109	0.201	−0.016	−0.168	0.136
15	Traffic accident	0.81	0.44	0.001	−0.045	−0.166	0.077	−0.018	−0.137	0.101
16	Residential fire	1.83	0.16	0.001	−0.075	−0.215	0.064	−0.033	−0.170	0.103
17	Domestic discord	1.39	0.25	0.001	−0.073	−0.238	0.091	−0.038	−0.199	0.123
18	Child abuse	0.82	0.44	0.001	−0.041	−0.196	0.114	−0.041	−0.193	0.111
19	Water accident	0.28	0.75	0.000	0.008	−0.143	0.159	−0.044	−0.192	0.104
20	War	2.41	0.09	0.002	0.139	−0.015	0.292	−0.048	−0.199	0.102
21	Lifestyle-related illness	2.05	0.13	0.001	−0.003	−0.133	0.127	−0.092	−0.219	0.035
22	Office building fire	4.48	0.01	0.003	−0.079	−0.231	0.074	−0.109	−0.259	0.040
23	Property offenses	3.38	0.03	0.002	−0.012	−0.142	0.119	−0.115	−0.243	0.014
24	Fire from home appliance	1.92	0.15	0.001	0.060	−0.088	0.207	−0.119	−0.263	0.026
25	Personal offenses	4.60	0.01	0.003	0.009	−0.135	0.153	**−0.159**	−0.300	−0.018
26	Alcohol	4.54	0.01	0.003	−0.011	−0.166	0.144	**−0.160**	−0.313	−0.008
27	Abnormal weather	10.49	<0.001	0.007	−0.084	−0.219	0.051	**−0.166**	−0.299	−0.034
28	Environmental pollution by chemicals	13.48	<0.001	0.009	−0.109	−0.238	0.021	**−0.167**	−0.294	−0.040
29	Nanotechnology	5.18	0.01	0.003	−0.009	−0.160	0.141	**−0.168**	−0.316	−0.020
30	Bullying in school	4.47	0.01	0.003	0.120	−0.039	0.278	**−0.193**	−0.348	−0.037
31	New infectious disease	13.95	<0.001	0.009	−0.079	−0.215	0.058	**−0.208**	−0.342	−0.075
32	Ultraviolet rays	14.53	<0.001	0.009	−0.078	−0.219	0.063	**−0.223**	−0.361	−0.085
33	Dioxin	13.38	<0.001	0.009	−0.060	−0.206	0.087	**−0.236**	−0.379	−0.092
34	Endocrine disruptor	20.57	<0.001	0.013	−0.111	−0.254	0.031	**−0.256**	−0.395	−0.116
35	Medical malpractice	20.73	<0.001	0.013	−0.100	−0.239	0.039	**−0.258**	−0.395	−0.122
36	BSE	29.38	<0.001	0.019	**−0.230**	−0.384	−0.076	**−0.258**	−0.409	−0.107
37	Medicinal side effect	15.86	<0.001	0.010	−0.044	−0.185	0.097	**−0.259**	−0.397	−0.120
38	Airplane accident	8.64	<0.001	0.006	**0.192**	0.032	0.352	**−0.264**	−0.420	−0.107
39	Tobacco	12.28	<0.001	0.008	−0.040	−0.204	0.125	**−0.268**	−0.429	−0.107
40	Accidental poisoning by home boiler	15.42	<0.001	0.010	−0.051	−0.207	0.106	**−0.282**	−0.435	−0.128
41	Terrorism	57.58	<0.001	0.036	**0.697**	0.541	0.853	**−0.287**	−0.440	−0.133
42	AIDS	15.71	<0.001	0.010	−0.071	−0.240	0.098	**−0.297**	−0.462	−0.131
43	Oil depletion	60.79	<0.001	0.038	**−0.335**	−0.476	−0.194	**−0.309**	−0.447	−0.171
44	Natural food additives	19.80	<0.001	0.013	−0.021	−0.165	0.123	**−0.311**	−0.453	−0.170
45	Asbestos	22.48	<0.001	0.014	−0.116	−0.279	0.047	**−0.319**	−0.479	−0.159
46	Mislabeling of food	27.99	<0.001	0.018	−0.070	−0.215	0.074	**−0.344**	−0.486	−0.203
47	GMO	24.61	<0.001	0.016	0.010	−0.135	0.155	**−0.366**	−0.509	−0.224
48	Indoor chemicals	32.13	<0.001	0.020	−0.036	−0.175	0.104	**−0.379**	−0.516	−0.242
49	Global warming	51.90	<0.001	0.033	−0.075	−0.200	0.051	**−0.414**	−0.537	−0.290
50	Chemical food additives	39.63	<0.001	0.025	0.021	−0.113	0.154	**−0.432**	−0.563	−0.301
51	Agrochemicals	57.77	<0.001	0.036	−0.078	−0.223	0.068	**−0.513**	−0.656	−0.370

Notes: *F*—*F* value; *p*—level of statistical significance; Partial η^2^—effect size of simple main effects; CI—confidence intervals; LL—Lower limit; UL—Upper limit; Positive values in difference scores indicate increases in anxiety and negative values indicate decreases in anxiety; Values in bold indicate significant results; Items were sorted in descending order of differences in anxiety scores between 2012 and 2008.
